# Characterizing Pet Acquisition and Retention During the COVID-19 Pandemic

**DOI:** 10.3389/fvets.2021.781403

**Published:** 2021-11-18

**Authors:** Christy L. Hoffman, Melissa Thibault, Julie Hong

**Affiliations:** ^1^Department of Animal Behavior, Ecology and Conservation, Canisius College, Buffalo, NY, United States; ^2^Strategy and Research, The American Society for the Prevention of Cruelty to Animals, New York, NY, United States; ^3^Communications, The American Society for the Prevention of Cruelty to Animals, New York, NY, United States

**Keywords:** COVID-19, pet ownership, pet acquisition, pet retention, companion animal, dog, cat

## Abstract

In March 2020, Americans began experiencing numerous lifestyle changes due to the COVID-19 pandemic. Some reports have suggested that pet acquisition and ownership increased during this period, and some have suggested shelters and rescues will be overwhelmed once pandemic-related restrictions are lifted and lifestyles shift yet again. In May 2021, the ASPCA hired the global market research company Ipsos to conduct a general population survey that would provide a more comprehensive picture of pet ownership and acquisition during the pandemic. Although pet owners care for a number of species, the term pet owner in this study specifically refers to those who had dogs and/or cats. One goal of the survey was to determine whether data from a sample of adults residing in the United States would corroborate findings from national shelter databases indicating that animals were not being surrendered to shelters in large numbers. Furthermore, this survey gauged individuals' concerns related to the lifting of COVID-19 restrictions, and analyses examined factors associated with pet owners indicating they were considering rehoming an animal within the next 3 months. The data showed that pet ownership did not increase during the pandemic and that pets may have been rehomed in greater numbers than occurs during more stable times. Importantly, rehomed animals were placed with friends, family members, and neighbors more frequently than they were relinquished to animal shelters and rescues. Findings associated with those who rehomed an animal during the pandemic, or were considering rehoming, suggest that animal welfare organizations have opportunities to increase pet retention by providing resources regarding pet-friendly housing and affordable veterinary options and by helping pet owners strategize how to incorporate their animals into their post-pandemic lifestyles.

## Introduction

In March 2020, quarantine recommendations were enacted in the United States due to the novel coronavirus SARS-CoV-2, which causes coronavirus disease 2019 (COVID-19). Several studies have reported that as many businesses shifted their employees to remote, work-from-home schedules, public demand for acquiring, or fostering a new pet grew (1–4). This apparent increase in demand likely was driven by a number of factors, including calls from animal welfare organizations to help “clear the shelters” (5, 6). With so many businesses, including restaurants and gyms, shut down during the early months of the pandemic and many employees working from home, individuals, and families had more time to spend at home with their pets. Furthermore, dog walking remained an accessible form of physical activity, and people may have sought out pets to reduce levels of stress, anxiety, and loneliness caused by the pandemic and the lifestyle changes it necessitated.

Despite people commonly spending more time at home during the pandemic and many reports indicating that the demand for pets grew as a result of COVID-19, shelter data shows that raw numbers of adoptions from shelters and rescues were actually lower in 2020 than in 2019 (7, 8). Furthermore, data collected by the American Pet Products Association (APPA) in December 2020 and March 2021 indicate that the percentage of households with cats decreased from 38% in their 2017–2018 report to 35% (9). The APPA reported that the prevalence of dog ownership, however, increased from 48% of households to 54% of households during that same period.

Data from the APPA's 2017–2018 national survey characterize pet acquisition trends leading up to the COVID-19 pandemic (10). Thirty-four percent of dogs and 37% of cats were adopted from a shelter or rescue. Furthermore, 25% of dogs and 26% of cats came from friends and relatives, and 29% of dogs and 5% of cats came from breeders and pet stores.

Some pandemic-related factors may have made pet acquisition and retention more challenging. During the early months of the pandemic, pet owners commonly expressed concerns about the availability and cost of veterinary care (11–13). A large-scale survey of pet owners in the US conducted during spring and summer 2020 reported that pet owners expressed difficulties securing pet supplies and dealing with their pet's behavioral issues (14). Participants in that survey also had concerns about what would happen to their pet if they became incapacitated with COVID-19, and they indicated they were preoccupied with managing familial and professional responsibilities and adjusting to working from home. Some pet owners who worked from home reported their pets created disruptions during the workday (14, 15), and about 9% of dog owners in one study reported their dogs displayed more attention-seeking behaviors during the pandemic (3).

Even with the challenges associated with pet-keeping during the pandemic, national data sources, including Shelter Animals Count in the US and Yad4 in Israel, did not show any noteworthy uptick in animal shelter intake or surrender rates in 2020 (3, 7). Indeed, in the US shelter intake numbers actually decreased from 2019 to 2020 (7). Additionally, national data aggregated from the shelter software system PetPoint indicated that in March and April 2021, cat intake numbers were merely approaching the range they were at during March and April 2019, while dog intake numbers remained uncharacteristically low (16). A number of factors contributed to reductions in shelter intakes, which thereby reduced the number of animals available for adoption. For instance, shelters commonly restricted intakes to animals in serious need and required that individuals make appointments to relinquish their animals. Additionally, the pandemic reduced the number of transports that moved dogs and cats from crowded shelters in one part of the country to less crowded ones elsewhere (17). Prior to the pandemic, robust transport programs helped ensure shelters featured a variety of dogs and cats for adoption, even in areas where local shelter intake rates were low (18).

Reductions in shelter intakes during the pandemic do not necessarily mean that overall rehoming rates were lower during this period. Animal shelters and rescues are not the only places where people turn when they can no longer keep their pets. In the APPA's 2017–2018 national survey of pet owners, over 70% of dog and cat owners indicated they would rehome their animals with a friend or family member if they could no longer keep them (10). Similarly, a study examining the re-homing of dogs and cats prior to the pandemic reported that while 36% were taken to a shelter, 37% were given to a friend or family member (19).

With shelter and rescue adoptions and intakes representing only part of the full picture of pet acquisitions and rehoming, in May 2021, the ASPCA hired the global market research company Ipsos to conduct a general population survey. The goals of the survey included the following: (1) To provide a more comprehensive picture of dog and cat ownership and acquisition during the COVID-19 pandemic; (2) To determine whether data from a sample of adults residing in the US would corroborate findings from national shelter databases indicating that dogs and cats, including those acquired during the pandemic, were not being surrendered to shelters in large numbers; (3) To gauge individuals' concerns associated with the lifting of COVID-19 restrictions and to assess whether any of these concerns, as well as demographic factors, were associated with dog and cat owners indicating they were considering rehoming a pet within the next 3 months.

## Methods

A national sample of 10,044 individuals 18 years and older residing in the continental United States, Alaska, and Hawaii were interviewed online in English as part of an omnibus poll conducted by Ipsos (ipsos.com) between May 17-June 1, 2021. To recruit participants, Ipsos used address-based sampling. If a household did not have the internet, Ipsos provided participants with free internet access and a tablet so they could complete the survey. Forty-one individuals were excluded from analyses due to inconsistencies in their responses, leaving a sample of 10,003 individuals. As the ASPCA received no identifying information about survey participants from Ipsos, the Advarra Institutional Review Board (IRB) deemed that the project (protocol number Pro00056183) did not meet the Department of Health and Human Service's definition of human subjects research under 45 CFR 46 and, therefore, did not require IRB oversight.

The survey included questions about the ownership of dogs and cats (referenced as “pets” or “animals” from here on) prior to March 2020, whether individuals owned any of these animals at the time of the survey, whether they had rehomed any animals since March 2020, and whether they were considering rehoming any animals within the upcoming 3 months. In addition, the survey asked individuals whether they had acquired and/or fostered animals during the pandemic. Those who indicated they had acquired an animal were then asked from where the animal was sourced (e.g., from a breeder; from a shelter or rescue organization; from an individual, friend, family member, or neighbor) and whether they still had the animal. If they no longer had the animal, they were asked where the animal was (e.g., with a friend, family member, or neighbor; at a shelter or rescue; deceased; lost). All participants were asked about their current work status (e.g., employed and working fully remotely, temporarily; employed and working fully remotely, permanently; employed and working fully away from home). In addition, participants were asked to rate on a 5-point Likert scale their degree of concern in relation to 7 statements (e.g., “I'm worried about being able to afford veterinary care for my animal”; “I'm worried about my employment and job security”; “I'm worried that I may not be able to stay in my home”). A score of 1 indicated no concern at all, and 5 indicated extreme concern. All participants provided demographic information, including their gender, age, race, household income, education level, and presence of children in the household. As an omnibus survey, the questionnaire also included questions from other Ipsos clients. The questions described above are the ones for which the ASPCA received data. The questionnaire has been included in the Supplementary Materials.

### Data Analysis

All analyses were conducted using R version 3.6.3 (20). Given the descriptive nature of the data, univariate analyses were comprised of frequency calculations and chi-square tests. When a chi-square test had more than 1 degree of freedom, a False Discovery Rate (FDR) *post-hoc* test was performed. Responses to the 7 questions regarding concerns were categorized as either no to low concern or moderate to high concern. That is, individuals who rated their level of concern as 1 or 2 were categorized as having no to low concern, and those who rated their level of concern as 3–5 were categorized as having moderate to high concern.

Binomial logistic regression modeling using the “lme4” package version 1.1–26 (21) assessed factors associated with acquiring an animal during the pandemic. The model contained demographic factors, including participant gender, age (18–34, 35–54, 55 years and older), whether there were children in the home, race (White, Asian American/Pacific Islander, Black or African American, Hispanic or Latino), household income (< $50,000, $50,000–$100,000, >$100,000), region of the country (South, Northeast, Midwest, West), community type (rural, suburban, or urban), and whether individuals had pets (i.e., dogs and/or cats) in the household prior to March 2020. The ANOVA function tested whether this model had a significantly better fit than the null model. The “car” package version 3.0–10 (22) was used to test for multicollinearity.

The same process was repeated to examine factors associated with having rehomed an animal during the pandemic. All factors included in the animal acquisition model were included in the rehoming model, with the exception that the factor examining pet ownership prior to March 2020 was replaced with a factor that divided pet owners (i.e., dog and cat owners) into the following three categories: had pets prior to March 2020 but did not acquire pets during the pandemic; had pets prior to March 2020 and acquired pets during the pandemic; and did not have pets prior to March 2020 but acquired pets during the pandemic.

Hierarchical binomial logistic regression was used to examine factors associated with whether those who had animals at the time of the survey were considering relinquishing them within the upcoming 3 months. All predictors described in the rehoming model were included in Model 1. Model 2 included the variables in Model 1, as well as the degree of participants' concerns regarding employment and job security, housing security, financial security, ability to afford veterinary care, pet behavior, having time to care for the pet, and pets impacting travel plans. Model 3 included all factors in Model 2 plus work status at the time the survey was completed (i.e., working from home or not). Finally, Model 4 included all variables in Model 3 and tested for an interaction between work status and household income. Individuals who indicated they did not need to work or selected “other” in response to the work status question were not included in these four models. The ANOVA function tested whether each model had a significantly better fit than the prior model. If there were no significant differences between models, the model with the lowest Akaike information criterion (AIC) value was chosen as the final model.

## Results

The demographic characteristics of the sample are summarized in Table 1. Just over half (53%) of the participants were female. Twenty-seven percent of participants were between 18 and 34 years old; 36% were between 35 and 54 years old, and the remaining individuals (38%) were over 55 years old. Children under the age of 18 years were present in 24% of households. The racial identities most frequently represented in the sample were White (77%), Black or African American (8%), Hispanic or Latino (8%), and Asian American or Pacific Islander (4%). Thirty-eight percent of participants reported annual household incomes under $50,000, and 22% reported household incomes over $100,000. The sample included individuals from all regions of the US, and just over half (52%) of the participants resided in suburban communities. At the time the survey was completed, 27% of participants were working fully away from home, 26% were working fully remotely, and 7% were working partly from home and partly away from home. Additionally, 13% were unemployed, and 19% were retired.

**Table 1:** Description of the survey sample.

	***N* (%)**
**Gender**	
Male	4,668 (46.7%)
Female	5,335 (53.3%)
**Age**	
18–34	2,674 (26.7%)
35–54	3,548 (35.5%)
55+	3,781 (37.8%)
**Children in household**	
Yes	2,358 (23.6%)
No	7,645 (76.4%)
**Race**	
Asian American/Pacific Islander	419 (4.2%)
Black or African American (not Hispanic or Latino)	837 (8.4%)
Hispanic or Latino	791 (7.9%)
Native American, Alaska Native, Aleutian	120 (1.2%)
White (not Hispanic or Latino)	7,666 (76.6%)
Other	79 (0.8%)
Prefer not to answer	91(0.9%)
**Household income**	
< $50,000	3,765 (37.6%)
$50,000–$100,000	4,063 (40.6%)
More than $100,000	2,175 (21.7%)
**Region**	
Midwest	2,375 (23.7%)
Northeast	1,835 (18.3%)
South	3,788 (37.9%)
West	2,005 (20.0%)
**Community type**	
Rural	2,391 (23.9%)
Suburban	5,177 (51.8%)
Urban	2,435 (24.3%)
**Work status at time of survey (percentages based on 6,562 individuals who acquired dogs and/or cats before and/or since March 2020)**	
Currently employed and working fully away from home	1,750 (26.7%)
Currently employed and working fully remotely, permanently	699 (10.7%)
Currently employed and working fully remotely, temporarily	1,026 (15.6%)
Currently employed and working partly remotely, partly away from home	459 (7.0%)
Currently unemployed	853 (13.0%)
Retired	1,242 (18.9%)
I don't need to work	194 (3.0%)
Other	339 (5.2%)

*Unless otherwise noted in the table, the percentages are based on the total sample of 10,003 participants*.

Of note, the likelihood of working from home fully or part-time at the time of the survey was significantly higher for those with household incomes >$100,000 compared to those in either of the lower-income categories [χ^2^_(2)_ = 244.27, *p* < 0.001]. Thirty-three percent of those with household incomes over $100,000 reported working from home, whereas 21% of those with household incomes between $50,000 and $100,000 and 16% of those with household incomes < $50,000 reported working from home. Supplementary Table 1 provides the breakdown of the numbers and percentages of individuals within the categories included in Table 1 who had acquired an animal since March 2020, rehomed an animal since March 2020, and were considering rehoming an animal in the upcoming 3 months.

As depicted in Table 2, prior to March 2020, 47% of participants had dogs, and 33% had cats. At the time of the survey, 45% had dogs, and 33% had cats. This suggests the proportions of households with dogs and/or cats did not increase from prior to the pandemic to May 2021. Moreover, only 4% of individuals who did not have dogs prior to the pandemic had dogs at the time of the survey, and 8% who had dogs prior to the pandemic no longer had them. Similarly, 3% of individuals who did not have cats prior to the pandemic had cats at the time of the survey, and 8% who had cats prior to the pandemic no longer had them. Table 2 also includes the proportion of pet owners who expressed moderate to high concern in response to questions related to the lifting of COVID-19 restrictions. As can be seen in the table, these concerns were shared by many pet owners. Notably, 57% of pet owners were concerned about their financial security, and 45% were concerned about their ability to afford veterinary care.

**Table 2:** Pet ownership statistics.

	***N* (%)**
**Pets pre-pandemic**	
Dogs	4,699 (47.0%)
Cats	3,329 (33.3%)
Dogs and/or cats	6,342 (63.4%)
**Pets acquired during pandemic**	
Dogs	1,284 (12.8%)
Cats	924 (9.2%)
Dogs and/or cats	1,901 (19.0%)
**Fostered animals during pandemic**	180 (1.8%)
**Rehomed pets since March 2020 (percentages based on 4,905 dog owners, 3,526 cat owners, and 6,562 dog and/or cat owners)**	
Dogs	508 (10.4%)
Cats	385 (10.9%)
Dogs and/or cats	783 (11.9%)
**Pets acquired during pandemic still with owner (percentages based on 1,284 dog owners, 924 cat owners, and 1,901 dog and/or cat owners)**	
Dogs	1,160 (90.3%)
Cats	799 (86.5%)
Dogs and/or cats	1,671 (87.9%)
**Pets at time of survey**	
Dogs	4,524 (45.2%)
Cats	3,265 (32.6%)
Dogs and/or cats	6,175 (61.7%)
**Considering relinquishing pet in next 3 months (percentages based on 4,524 dog owners, 3,265 cat owners, and 6,175 individuals with dogs and/or cats)**	
Dogs	360 (8.0%)
Cats	276 (8.5%)
Dogs and/or cats	532 (8.6%)
**Pet owners who indicated a moderate to high level of concern (percentages based on 6,175 individuals who owned dogs and/or cats at the time of the survey)**	
I'm worried about my employment and job security.	2,416 (39.1%)
I'm worried that I may not be able to stay in my home.	1,865 (30.2%)
I'm worried about my financial security.	3,492 (56.6%)
I'm worried about being able to afford veterinary care for my animal.	2,799 (45.3%)
I'm worried my animal will have behavior problems as a result of a change in schedule.	2,067 (33.5%)
I'm worried I won't have as much time to care for and/or spend with my animal.	2,257 (36.6%)
I'd like to travel more and I feel limited by my animal.	2,836 (45.9%)

*Unless otherwise noted in the table, the percentages are based on the total sample of 10,003 participants*.

### Animal Acquisition During the Pandemic

Two percent of individuals reported having fostered an animal since March 2020, 13% had acquired one or more dogs, and 9% had acquired one or more cats (Table 2). Pets acquired during the pandemic came from a variety of sources (Table 3). The most common sources from which dogs were acquired included breeders (36%); individuals, including friends, family members, and neighbors (24%); shelters or rescues (22%); and pet stores (14%). Cats most commonly came from individuals, including friends, family members, and neighbors (33%); shelters or rescues (24%); breeders (19%); and pet stores (15%).

**Table 3:** Sources of the 1,284 dogs and 924 cats acquired during the pandemic.

	**Dog**	**Cat**
	***N* (%)**	***N* (%)**
From a breeder (did not see where the animal was raised)	126 (9.8%)	61 (6.6%)
From a breeder (saw where the animal was raised)	336 (26.2%)	111 (12.0%)
From a pet store	183 (14.3%)	136 (14.7%)
From a shelter or rescue organization	288 (22.4%)	217 (23.5%)
From an individual, friend, family member, or neighbor (for free)	166 (12.9%)	242 (26.2%)
From an individual, friend, family member, or neighbor (purchased)	148 (11.5%)	64 (6.9%)
Other	37 (2.9%)	93 (10.1%)

Given similarities in patterns observed regarding dog and cat acquisition and ownership during the pandemic, dogs and cats were combined in the model that tested which factors were associated with having acquired an animal during the pandemic. They were also combined in models examining factors associated with (1) having rehomed an animal during the pandemic and (2) considering rehoming an animal in the upcoming 3 months. The pet acquisition model had a significantly better fit than the null model (Δ*-2LL* = 1542.6, Δ*df* = 15, *p* < 0.001). As Table 4 depicts, numerous factors were associated with having acquired an animal since March 2020. Females were less likely to have acquired an animal than males (*OR* = 0.65), and those in the older two age groups were less likely to have acquired an animal than those 18–34 years (35–54: *OR* = 0.56, 55+: *OR* = 0.24). Those with children in the household were 1.83 times more likely to have acquired an animal than those without, while those with incomes $50,000–$100,000 were less likely to have acquired an animal than those in the lowest income group (*OR* = 0.87). Compared to White participants in the sample, those of Latino or Hispanic descent were 1.29 times more likely to have acquired an animal. In addition, compared to those living in the South, those living in the Midwest, Northeast, and West were significantly less likely to have acquired an animal (Midwest: *OR* = 0.77, Northeast: *OR* = 0.73, West: *OR* = 0.78). Additionally, individuals living in rural and urban areas were significantly more likely to have acquired an animal than those living in the suburbs (rural: *OR* = 1.26, urban: *OR* = 1.70). Finally, those who had dogs and/or cats prior to the pandemic were 4.71 times more likely to have acquired an animal than those who did not.

**Table 4:** Results of binary logistic regression model assessing factors associated with having acquired a dog and/or cat during the pandemic.

**Predictors**	**Model**		
	**Odds ratios**	**CI**	***p***
(Intercept)	0.14	0.11–0.17	**<0.001**
**Gender (ref: Male)**			
Female	0.65	0.58–0.72	**<0.001**
**Age category (ref: 18**–**34 years)**			
35–54 years	0.56	0.49–0.63	**<0.001**
55+ years	0.24	0.21–0.29	**<0.001**
**Children in household (ref: No)**			
Yes	1.83	1.61–2.07	**<0.001**
**Race (ref: White, not Hispanic or Latino)**			
Asian American/Pacific Islander	0.84	0.62–1.12	0.244
Black or African American (not Hispanic or Latino)	1.11	0.90–1.35	0.321
Hispanic or Latino	1.29	1.07–1.55	**0.007**
**Household income (ref:** ** < $50,000)**			
$100,000+	1.04	0.89–1.21	0.611
$50,000–$100,000	0.87	0.77–0.99	**0.038**
**Region (ref: South)**			
Midwest	0.77	0.66–0.89	**<0.001**
Northeast	0.73	0.62–0.86	**<0.001**
West	0.78	0.67–0.92	**0.002**
**Community type (ref: Suburban)**			
Rural	1.26	1.10–1.45	**0.001**
Urban	1.70	1.49–1.94	**<0.001**
**Dog/cat ownership (ref: Did not have animal prior to March 2020)**			
Had animals prior to March 2020	4.71	4.04–5.52	**<0.001**
Observations	9,713		
−2LL	7,944.8		
AIC	7,676.8		
R^2^ Tjur	0.160		

*The bold values represent p-values less than 0.05*.

### Outcomes for Animals Acquired During the Pandemic

Of the animals acquired during the pandemic, 90% of dogs and 87% of cats were still with their owners (Table 2). Details regarding outcomes for animals no longer with their owners at the time of the survey are provided in Table 5. Half of the dogs in this situation went to a friend, family member, or neighbor; 17% went to a shelter or rescue; 13% had died; and 12% were sold. Similarly, 37% of cats went to a friend, family member, or neighbor; 26% went to a shelter or rescue; 13% had died; and 13% were sold.

**Table 5:** Outcomes regarding the 124 dogs and 125 cats acquired during the pandemic that are no longer with their owners.

	**Dog**	**Cat**
	***N (%)***	***N (%)***
I sold my animal	15 (12.1%)	16 (12.8%)
My animal died	16 (12.9%)	16 (12.8%)
My animal got lost	7 (5.6%)	8 (6.4%)
They are at a shelter or rescue	21 (16.9%)	33 (26.4%)
They are with a friend, family or neighbor	62 (50.0%)	46 (36.8%)
Other	3 (2.4%)	6 (4.8%)

### Factors Associated With Rehoming a Dog or Cat During the Pandemic

Since March 2020, 12% of pet owners reported having rehomed a dog and/or cat (Table 2). The binomial logistic model that tested which factors were associated with having rehomed an animal during the pandemic had a significantly better fit than the null model (Δ*-2LL* = 1098.3, Δ*df* = 16, *p* < 0.001). As Table 6 shows, females were less likely to have rehomed an animal than males (*OR* = 0.47). Individuals in the older two age categories were less likely to have rehomed an animal than those aged 18–34 (35–54: *OR* = 0.63, 55+: *OR* = 0.40). Having children in the household increased the odds of having rehomed an animal (*OR* = 1.78). Additionally, compared to White participants, Black participants were 1.42 times more likely to have rehomed an animal, and those who reported household incomes >$50,000 were less likely to have rehomed an animal than those with household incomes below $50,000 ($50,000–$100,000: OR = 0.70, $100,000+: OR = 0.77). Individuals living in urban communities were more likely to have rehomed an animal than those residing in suburban communities (*OR* = 1.80), and those living in the Northeast were less likely to have rehomed an animal than those living in the South (*OR* = 0.75). Compared to dog and cat owners who had not acquired dogs and/or cats since March 2020, those who had acquired dogs and/or cats both prior to March 2020 and since March 2020 were 7.18 times more likely to have rehomed a pet, and those who did not have dogs or cats prior to March 2020 and had acquired one or more since March 2020 were 3.31 times more likely to have done this.

**Table 6:** Results of binary logistic regression model assessing factors associated with rehoming a dog and/or cat during the pandemic.

**Predictors**	**Model**		
	**Odds ratios**	**CI**	***p***
(Intercept)	0.09	0.07–0.12	**<0.001**
**Gender (ref: Male)**			
Female	0.47	0.40–0.56	**<0.001**
**Age category (ref: 18–34 years)**			
35–54 years	0.63	0.52–0.76	**<0.001**
55+ years	0.40	0.30–0.53	**<0.001**
**Children in household (ref: No)**			
Yes	1.78	1.47–2.16	**<0.001**
**Race (ref: White, not Hispanic or Latino)**			
Asian American/Pacific Islander	0.76	0.44–1.26	0.310
Black or African American (not Hispanic or Latino)	1.42	1.06–1.90	**0.016**
Hispanic or Latino	1.28	0.98–1.67	0.069
**Household income (ref:** ** < $50,000)**			
$100,000+	0.77	0.61–0.98	**0.032**
$50,000–$100,000	0.70	0.57–0.86	**0.001**
**Region (ref: South)**			
Midwest	0.98	0.78–1.22	0.857
Northeast	0.75	0.58–0.97	**0.029**
West	0.83	0.65–1.06	0.132
**Community type (ref: Suburban)**			
Rural	1.18	0.94–1.48	0.151
Urban	1.80	1.47–2.19	**<0.001**
**Dog/cat ownership (ref: Acquired animals before March 2020 but**			
**not since)**			
Animals acquired before March 2020 and during pandemic	7.18	5.97–8.67	**<0.001**
Animals acquired during pandemic but not before	3.31	2.21–4.86	**<0.001**
Observations	6,375		
−2LL	3588.0		
AIC	3622.0		
R^2^ Tjur	0.206		

*The bold values represent p-values less than 0.05*.

### Factors Associated With Potential Future Relinquishment

Approximately 9% of participants with dogs and/or cats indicated they were considering rehoming an animal in the upcoming 3 months (Table 2). Of the four models that assessed factors associated with participants indicating they were considering rehoming an animal, Model 3 had the best fit. The table comparing all four models has been included as Supplementary Table 2.

According to Model 3, females were less likely to be considering rehoming than males (*OR* = 0.48), and individuals over 55 years of age were less likely to be considering rehoming than individuals 18–34 years (*OR* = 0.65). Having children in the household increased the odds of individuals indicating they were considering rehoming (*OR* = 1.79). There was no association between race and likelihood of considering rehoming. Individuals with household incomes >$100,000 were 1.42 times more likely to indicate they were considering rehoming an animal than those with incomes < $50,000. Additionally, those in rural and urban areas were more likely to indicate they were considering rehoming an animal than those in suburban areas (rural: *OR* = 1.59, urban: *OR* = 1.92), but no regional differences were observed. Compared to dog and cat owners who had not acquired dogs and/or cats since March 2020, those who had acquired dogs and/or cats both prior to March 2020 and since March 2020 were 4.80 times more likely to indicate they were considering rehoming. Furthermore, those who did not have dogs or cats prior to March 2020 and had acquired one or more since March 2020 were 3.02 times more likely to indicate this. Individuals with concerns regarding housing security were more likely to say they were considering rehoming (*OR* = 1.84), as were individuals with concerns regarding being able to afford veterinary care (*OR* = 1.79). Moreover, working from home was associated with being more likely to indicate one was considering rehoming (*OR* = 1.90).

## Discussion

Although several prior studies have indicated that pet acquisitions increased during the COVID-19 pandemic (1–4), the national survey data collected in May 2021 did not show that ownership of dogs or cats had increased. While the percentage of survey participants who indicated having dogs and/or cats prior to March 2020 closely matches dog and cat ownership statistics reported in the APPA's 2017–2018 national survey of pet owners (10), we observed a slight decline in those numbers from the period prior to March 2020 to when survey data were collected in May 2021.

The APPA's 2021–2022 survey, for which data were collected in December 2020 and March 2021, showed that cat ownership had decreased since the APPA's 2017–2018 report (9). However, their data indicated the percentage of households with dogs had increased. That we found a slight decrease in dog ownership and the APPA found an increase from their 2018–2019 survey to their 2021–2022 survey is puzzling. It is possible that the timing of data collection contributed to the differences between our survey and the APPA's most recent survey. When the 2021–2022 APPA data were collected, vaccinations to protect against COVID-19 were not widely available, and many social distancing protocols were still in place. By the time our survey data were collected at the end of May 2021, vaccinations were readily available to adults across the US, and much of the population had begun returning to pre-pandemic school, work, and social routines. The shift in lifestyle may have made dog keeping more challenging and led to an uptick in rehoming. The timing of these surveys may also contribute to why we found that 10% of dog owners and 11% of cat owners had rehomed an animal during the pandemic, whereas the APPA's 2021–2022 report concluded that COVID-19 had caused 6% of dog owners and 7% of cat owners to rehome their animals.

The pandemic appears to have resulted in some changes to how pets were acquired. This may have been due to reductions in the overall number of animals available for adoption during the pandemic, as well as to the public's limited accessibility to shelters during this period. In comparison to the APPA's 2017–2018 report (10), the proportion of cats and dogs acquired from shelters and rescues decreased during the pandemic while the proportion of cats and dogs acquired from pet stores and breeders increased. As fewer animals were relinquished to shelters during the pandemic (16), the demand for shelter animals may have outpaced the numbers of animals available for adoption. Slowdowns in shelter and rescue operations due to pandemic-related restrictions and protocols also may have contributed to the decline in numbers of animals taken into and adopted from shelters and rescues. Adoption facilities around the country limited community members' opportunities to relinquish animals, closed their facilities to walk-in visitors, and required that both intakes and adoptions occur via appointment.

Many of the factors that were associated with having acquired an animal during the pandemic aligned with the APPA's 2017–2018 findings regarding characteristics of pet owners (10). For instance, the organization reported that pet ownership was more common among younger individuals and in households that included children. We found that individuals within these categories also were more likely to have acquired a dog and/or cat during the pandemic.

The regional differences we observed in pet acquisition during the pandemic may relate to regional differences in the availability of animals. Animal shelters in the South tend to have more animals in need of rehoming than shelters in other parts of the US (23). Furthermore, many of the transports that, prior to the pandemic, had moved dogs and cats from crowded shelters in the South to less crowded ones elsewhere were halted (17). Additionally, the South consistently had less stringent COVID-19 mitigation strategies than most other US regions (24), and so this is yet another factor that may have made it easier to acquire a new pet in the South during the pandemic.

Pet acquisition rates reported in this survey also differed by gender. That is, men were more likely to report having acquired a pet during the pandemic than women. This disparity may relate to systemic gender inequalities that contributed to the pandemic's differential impacts on men and women. During this period, more women than men lost their jobs, worked essential jobs that increased their risk of exposure to COVID-19 and its associated stressors, and experienced significant increases in familial responsibilities (25, 26). Furthermore, the switch to remote work that many experienced during the pandemic may have impacted men and women differently. A prior study concluded that men found telecommuting to be more restorative than did women (27). Additionally, women who telework commonly report they have less leisure time (28). Thus, men may have viewed pandemic-related changes as increasing their capacity to care for a pet while women may have experienced the opposite. These perceptions may have been short-lived, though. More men than women reported rehoming an animal during the pandemic, and men were more likely to indicate they were planning to do so in the upcoming 3 months.

The strongest predictor of having acquired an animal during the pandemic was pet ownership prior to the pandemic. Specifically, those who had dogs and/or cats prior to March 2020 were significantly more likely to have acquired one during the pandemic than were those who entered the pandemic without a pet. This aligns with prior research suggesting that the decision to acquire a pet commonly is influenced by prior pet ownership (29, 30).

Ninety percent of individuals who acquired a dog during the pandemic still had their animal, as did 87% of those who acquired a cat. Moreover, 88% of pet owners, including those who acquired their pets before the pandemic, reported they did not rehome an animal during this time period. While these findings indicate that pets were not being rehomed in massive numbers, the percentages of animals that were rehomed may be larger than is typical during economically stable times. A national survey conducted by Weiss et al. prior to the pandemic found that only 6% of individuals reported having rehomed a dog and/or cat within the 5-year period spanning 2009–2014 (19). Like the COVID-19 pandemic, the Great Recession of 2008 had a destabilizing effect on the economy and countless Americans' lives. A comparison of shelter intake records in the Chicago area prior to and during the recession showed an uptick in the numbers of dogs relinquished during the recession, although this trend was not observed for cats (31). Importantly, however, that study did not include the number of animals that were rehomed without ever entering the shelter system and so likely underestimated the number of animals rehomed during that challenging period. As was observed in the current study, Weiss et al. found that pet owners commonly rehome their animals with friends, family members, and neighbors (19).

Individuals who did acquire new animals during the pandemic were more likely to report they had rehomed an animal during the pandemic than were pet owners who did not acquire new animals during that period. In some cases, a participant may have found themselves needing to rehome their pre-pandemic pet due to pandemic-inspired difficulties (e.g., job loss, illness) and then acquired a new one as their situation improved. Another potential explanation for this finding is that those who acquired a pet during the pandemic may have had trouble securing the resources and training assistance they needed to keep their animals. In a large-scale survey of pet owners conducted in the US during spring and summer 2020, participants expressed difficulties obtaining pet supplies and dealing with their pets' behavioral issues (14). As our survey did not ask participants about the timing of pet rehoming and acquisition, we were unable to determine how often rehoming preceded vs. followed pet acquisition and whether the majority of pets rehomed were those acquired during the pandemic.

Participant age also was associated with the likelihood of rehoming an animal. Adults under 35 years old were more likely to have rehomed a pet during the pandemic than were older adults. Prior research on animal shelter relinquishment indicates that, even prior to the pandemic, younger adults were at greater risk of rehoming their pets than were older adults (32). This pattern may relate to the reality that younger adults disproportionately rent rather than own their housing and commonly face challenges finding pet-friendly housing (33). Even before the pandemic, the dearth of pet-friendly, affordable housing was a leading cause of pet relinquishment (19, 34–36). The pandemic would have made access to pet-friendly housing particularly difficult for young adults who lost their job or had their wages and/or work hours reduced.

Housing-related concerns may at least partially explain why Black participants were more likely to report having rehomed an animal during the pandemic than White participants. COVID-19 highlighted and exacerbated racial inequalities in the US (37), including longstanding racial inequalities in the availability of pet-friendly housing (34). Individuals belonging to racial or ethnic minority groups not only were more vulnerable to severe illness from COVID-19 (38) but also were disproportionately impacted by pandemic-related layoffs and reductions in wages and hours (39).

Analyses regarding which pet owners were considering rehoming an animal in the upcoming 3 months provided additional insights into challenges pet owners faced because of the pandemic. Compared to those not considering rehoming, these individuals were more likely to express a moderate to high degree of concern about housing security and their ability to afford veterinary care. This finding is in line with those from a recent report that 61% of pet owners polled within the US were very concerned about their finances over the upcoming year (40).

An unanticipated finding was that those with household incomes above $100,000 were more likely to indicate that they were considering rehoming a pet than those in the lowest income group, even though they were not more likely to have acquired a pet during the pandemic. This was true even after including in the model whether individuals were working from home (i.e., workplace location). Additionally, adding to the full main effects model an interaction term between household income and workplace location did not improve the model fit. This suggests that those in households with incomes >$100,000 may have had pet-related concerns that were independent of their workplace location and the other variables included in the model. It is possible individuals in this income category were anticipating bigger lifestyle changes with the lifting of COVID-19 restrictions compared to individuals in the lower-income group.

Although behavioral concerns were not a significant predictor in the model examining factors associated with considering rehoming, it is notable that one-third of pet owners surveyed in the current study were concerned about their pets developing behavioral problems. During periods of lockdown and working from home, pet owners indicated their pets were rarely alone, and they were worried about how pets would handle being left alone (41, 42). The breakdown in predictable pandemic-related routines, such as lunchtime dog walks, that occur as schedules revert back to what they were prior to March 2020 may result in pets expressing behaviors indicative of anxiety, boredom, and frustration (43). Symptoms of anxiety, boredom, and frustration may manifest as destructive behaviors and increased vocalizations and attention-seeking behaviors.

Findings regarding pet owners' concerns and which pet owners rehomed an animal during the pandemic, or were considering rehoming an animal, suggest that some individuals may need assistance planning how to incorporate their pets into their evolving lifestyle. To reduce the number of animals entering shelters, animal welfare organizations have devised numerous ways to help pet owners. Prior to the pandemic, many animal shelters and rescues already offered safety net programs, such as behavior helplines, preventative veterinary care clinics, and pet food pantries, to reduce relinquishments (44). Some animal welfare organizations also help individuals with housing-related concerns. Organizations, such as Pet Housing Help AZ in Arizona (https://pethousinghelpaz.org/) and Fulton County Animal Services in Georgia (https://www.fultonanimalservices.com/resources-services/pet-help), that offer short-term, free or low-cost pet housing options for pet owners in transition from one housing situation to another may prove especially important to reduce the numbers of animals in need of permanent rehoming. Shelters also can reduce intakes by helping owners rehome their animals without having to surrender them to the shelter. For example, shelters can maintain a website of pet profiles featuring animals that are currently in homes and need a new place to live (45). As of August 2021, 473 organizations had partnered with the coalition Human Animal Support Services, which has the mission of keeping people and their pets together (46). This suggests that many animal welfare organizations are prioritizing efforts that reduce the number of animals in need of rehoming.

### Strengths and Limitations

While the data reported herein represent findings from a large national sample of pet owners and non-pet owners across the US, the study did have some limitations. Ipsos, the company that collected the data, attempted to minimize sampling bias by using address-based sampling and provisioning those who needed it with internet access and a tablet so they could complete the survey. Nevertheless, the sample was not entirely representative of the US population. Based on 2020 US Census Bureau data (47), White participants were overrepresented in the sample while other racial groups were underrepresented. This may have been due in part to collecting responses solely from English-speaking individuals. US Census Bureau data also indicate that individuals in households earning over $100,000 per year were underrepresented in the sample while those in households earning $50,000–$100,000 were overrepresented (48). Moreover, the 2020 US census data show that the survey underrepresented households that included children under 18 years (49). Additionally, the total number of dogs and cats participants owned and/or rehomed prior to and during the pandemic is unknown, as was detailed information about these animals. Furthermore, the survey did not ask about household pets other than cats and dogs.

While some pandemic-related restrictions had been lifted when the data were collected in May 2021, many were still in effect. Consequently, it is hard to predict how pet ownership trends will change and how many individuals will actually rehome their animals once restrictions are lifted completely. When individuals express intentions regarding future behaviors, they commonly do not follow through with the planned behavior (50). In the case of rehoming pets, perhaps many of those who stated they were considering rehoming their pets will not actually do so; however, the percentages of individuals who have rehomed animals over the past year and who indicated they are considering rehoming pets suggest that some pet owners would benefit from pet retention resources and support. Despite the unknowns regarding future behavior, this study provided new insights into factors associated with both acquiring and rehoming dogs and cats during the COVID-19 pandemic.

## Conclusion

The COVID-19 pandemic presented unique challenges to pet acquisition and ownership, and while some reports have suggested that pet ownership increased, findings from this study reflect that pet ownership numbers are actually slightly below what they were prior to March 2020. The pandemic adversely affected housing security, thereby exacerbating challenges to maintaining a pet in the household. Moderate to high degrees of concern in this domain, as well as concerns about being able to afford veterinary care, increased the likelihood of participants indicating they were considering rehoming their animal in the near future. Other risk factors associated with individuals who were considering rehoming pets in the near future included having children in the home and working from home. These findings suggest that individuals who fall into these categories might benefit from the assistance of animal welfare organizations and animal care professionals who can help them strategize ways to meet their pets' physical and psychological needs as they transition into a post-pandemic lifestyle.

## Data Availability Statement

The raw data supporting the conclusions of this article will be made available by the authors, without undue reservation.

## Author Contributions

CH: formal analysis, methodology, writing—original draft and revision, and data curation. MT: conceptualization, investigation, and writing—review and editing. JH: conceptualization. All authors contributed to the article and approved the submitted version.

## Funding

This study was funded by the American Society for the Prevention of Cruelty to Animals.

## Conflict of Interest

The authors declare that the research was conducted in the absence of any commercial or financial relationships that could be construed as a potential conflict of interest.

## Publisher's Note

All claims expressed in this article are solely those of the authors and do not necessarily represent those of their affiliated organizations, or those of the publisher, the editors and the reviewers. Any product that may be evaluated in this article, or claim that may be made by its manufacturer, is not guaranteed or endorsed by the publisher.
