# Supplementary material for: Characterizing Pet Acquisition and Retention During the COVID-19 Pandemic
Source: Front Vet Sci. 2021 Nov 18;8:781403. doi: 10.3389/fvets.2021.781403 (PMC8637628; doi:10.3389/fvets.2021.781403)
Supplement: Supplementary file 1 [file Table_1.DOCX]

**Supplementary Table 1.** The numbers and percentages of individuals within the categories included in Table 1 who had acquired an animal since March 2020, rehomed an animal since March 2020, and were considering rehoming an animal in the upcoming three months. Unless otherwise noted in the table, the percentages are based on the total sample of 10003 participants.

|  | Acquired an Animal | |  | Rehomed an Animal | |  | Considering Rehoming | |
| --- | --- | --- | --- | --- | --- | --- | --- | --- |
|  | Yes | No |  | Yes | No |  | Yes | No |
|  | *N (%)* | *N (%)* |  | *N (%)* | *N (%)* |  | *N (%)* | *N (%)* |
| *Gender* |  |  |  |  |  |  |  |  |
| Male | 1027 (22.0%) | 3641 (78.0%) |  | 512 (17.2%) | 2457 (82.8%) |  | 389 (14.1%) | 2379 (85.9%) |
| Female | 874 (16.4%) | 4461 (83.6%) |  | 271 (7.5%) | 3322 (92.5%) |  | 143 (4.2%) | 3264 (95.8%) |
|  |  |  |  |  |  |  |  |  |
| *Age* |  |  |  |  |  |  |  |  |
| 18-34 | 855 (32.0%) | 1819 (68.0%) |  | 397 (19.5%) | 1634 (80.5%) |  | 238 (12.6%) | 1646 (87.4%) |
| 35-54 | 784 (22.1%) | 2764 (77.9%) |  | 310 (12.1%) | 2243 (87.9%) |  | 257 (10.6%) | 2179 (89.4%) |
| 55+ | 262 (6.9%) | 3519 (93.1%) |  | 76 (3.8%) | 1902 (96.2%) |  | 37 (2.0%) | 1818 (98.0%) |
|  |  |  |  |  |  |  |  |  |
| *Children in Household* |  |  |  |  |  |  |  |  |
| Yes | 780 (33.1%) | 1578 (66.9%) |  | 377 (20.3%) | 1484 (79.7%) |  | 305 (17.3%) | 1456 (82.7%) |
| No | 1121 (14.7%) | 6524 (85.3%) |  | 406 (8.6%) | 4295 (91.4%) |  | 227 (5.1%) | 4187 (94.9%) |
|  |  |  |  |  |  |  |  |  |
| *Race* |  |  |  |  |  |  |  |  |
| Asian American/Pacific Islander | 64 (15.3%) | 355 (84.7%) |  | 20 (10.2%) | 176 (89.8%) |  | 11 (5.9%) | 175 (94.1%) |
| Black or African American (not Hispanic or Latino) | 185 (22.1%) | 652 (77.9%) |  | 99 (24.2%) | 311 (75.8%) |  | 69 (19.0%) | 295 (81.0%) |
| Hispanic or Latino | 231 (29.2%) | 560 (70.8%) |  | 107 (19.1%) | 454 (80.9%) |  | 66 (12.7%) | 453 (87.3%) |
| Native American, Alaska Native, Aleutian | 20 (16.7%) | 100 (83.3%) |  | 9 (9.5%) | 86 (90.5%) |  | 3 (3.5%) | 84 (96.5%) |
| White (not Hispanic or Latino) | 1380 (18.0%) | 6286 (82.0%) |  | 541 (10.4%) | 4667 (89.6%) |  | 378 (7.7%) | 4559 (92.3%) |
| Other | 11 (13.9%) | 68 (86.1%) |  | 4 (8.9%) | 41 (91.1%) |  | 4 (10.0%) | 36 (90.0%) |
| Prefer not to answer | 10 (11.0%) | 81 (89.0%) |  | 3 (6.4%) | 44 (93.6%) |  | 1 (2.4%) | 41 (97.6%) |
|  |  |  |  |  |  |  |  |  |
| *Household Income* |  |  |  |  |  |  |  |  |
| Less than $50,000 | 720 (19.1%) | 3045 (80.9%) |  | 323 (13.6%) | 2057 (86.4%) |  | 164 (7.5%) | 2029 (92.5%) |
| $50,000 to $100,000 | 692 (17.0%) | 3371 (83.0%) |  | 250 (9.5%) | 2392 (90.5%) |  | 173 (6.9%) | 2343 (93.1%) |
| More than $100,000 | 489 (22.5%) | 1686 (77.5%) |  | 210 (13.6%) | 1330 (86.4%) |  | 195 (13.3%) | 1271 (86.7%) |
|  |  |  |  |  |  |  |  |  |
| *Region* |  |  |  |  |  |  |  |  |
| Midwest | 392 (16.5%) | 1983 (83.5%) |  | 164 (10.9%) | 1335 (89.1%) |  | 102 (7.3%) | 1300 (92.7%) |
| Northeast | 286 (15.6%) | 1549 (84.4%) |  | 106 (9.6%) | 995 (90.4%) |  | 84 (8.2%) | 938 (91.8%) |
| South | 875 (23.1%) | 2913 (76.9%) |  | 383 (14.5%) | 2255 (85.5%) |  | 260 (10.4%) | 2250 (89.6%) |
| West | 348 (17.4%) | 1657 (82.6%) |  | 130 (9.8%) | 1194 (90.2%) |  | 86 (6.9%) | 1155 (93.1%) |
|  |  |  |  |  |  |  |  |  |
| *Community Type* |  |  |  |  |  |  |  |  |
| Rural | 462 (19.3%) | 1929 (80.7%) |  | 169 (9.7%) | 1568 (90.3%) |  | 104 (6.3%) | 1544 (93.7%) |
| Suburban | 784 (15.1%) | 4393 (84.9%) |  | 267 (8.2%) | 2994 (91.8%) |  | 158 (5.1%) | 2913 (94.9%) |
| Urban | 655 (26.9%) | 1780 (73.1%) |  | 347 (22.2%) | 1217 (77.8%) |  | 270 (18.5%) | 1186 (81.5%) |
|  |  |  |  |  |  |  |  |  |
| *Work Status at Time of Survey (percentages based on 6562 individuals who acquired dogs and/or cats before and/or since March 2020)* |  |  |  |  |  |  |  |  |
| Currently employed and working fully away from home | NA | NA |  | NA | NA |  | 74 (4.4%) | 1601 (95.6%) |
| Currently employed and working fully remotely, permanently | NA | NA |  | NA | NA |  | 106 (15.9%) | 562 (84.1%) |
| Currently employed and working fully remotely, temporarily | NA | NA |  | NA | NA |  | 229 (23.4%) | 749 (76.6%) |
| Currently employed and working partly remotely, partly away from home | NA | NA |  | NA | NA |  | 48 (11.3%) | 377 (88.7%) |
| Currently unemployed | NA | NA |  | NA | NA |  | 40 (5.1%) | 742 (94.9%) |
| Retired | NA | NA |  | NA | NA |  | 19 (1.7%) | 1132 (98.3%) |
| I don't need to work | NA | NA |  | NA | NA |  | 5 (2.8%) | 174 (97.2%) |
| Other | NA | NA |  | NA | NA |  | 11 (3.5%) | 306 (96.5%) |
