# Supplementary material for: Characterizing Pet Acquisition and Retention During the COVID-19 Pandemic
Source: Front Vet Sci. 2021 Nov 18;8:781403. doi: 10.3389/fvets.2021.781403 (PMC8637628; doi:10.3389/fvets.2021.781403)
Supplement: Supplementary file 2 [file Table_2.DOCX]

**Supplementary Table 2.** Results of binary logistic regression model assessing factors associated with considering relinquishing dog and/or cat in the upcoming 3 months. The bolded Akaike information criterion (AIC) value indicates which model was best.

|  | **Model 1** | | | **Model 2** | | | **Model 3** | | | **Model 4** | | |
| --- | --- | --- | --- | --- | --- | --- | --- | --- | --- | --- | --- | --- |
| *Predictors* | *Odds Ratios* | *CI* | *p* | *Odds Ratios* | *CI* | *p* | *Odds Ratios* | *CI* | *p* | *Odds Ratios* | *CI* | *p* |
| (Intercept) | 0.04 | 0.02 – 0.05 | **<0.001** | 0.01 | 0.01 – 0.01 | **<0.001** | 0.01 | 0.00 – 0.01 | **<0.001** | 0.01 | 0.00 – 0.01 | **<0.001** |
| *Gender (Ref: Male)* |  |  |  |  |  |  |  |  |  |  |  |  |
| Female | 0.36 | 0.28 – 0.45 | **<0.001** | 0.46 | 0.36 – 0.58 | **<0.001** | 0.48 | 0.38 – 0.61 | **<0.001** | 0.48 | 0.38 – 0.61 | **<0.001** |
| *Age Category (Ref: 18-34 years)* |  |  |  |  |  |  |  |  |  |  |  |  |
| 35-54 years | 0.78 | 0.62 – 0.99 | **0.039** | 0.88 | 0.69 – 1.12 | 0.292 | 0.90 | 0.71 – 1.15 | 0.412 | 0.90 | 0.71 – 1.15 | 0.415 |
| 55+ years | 0.36 | 0.23 – 0.53 | **<0.001** | 0.57 | 0.37 – 0.86 | **0.008** | 0.65 | 0.42 – 0.99 | **0.050** | 0.65 | 0.42 – 0.99 | **0.048** |
| *Children in Household (Ref: No)* |  |  |  |  |  |  |  |  |  |  |  |  |
| Yes | 2.14 | 1.70 – 2.70 | **<0.001** | 1.86 | 1.46 – 2.37 | **<0.001** | 1.79 | 1.40 – 2.29 | **<0.001** | 1.79 | 1.40 – 2.28 | **<0.001** |
| *Race (Ref: White, not Hispanic or Latino)* |  |  |  |  |  |  |  |  |  |  |  |  |
| Asian American/Pacific Islander | 0.63 | 0.31 – 1.19 | 0.182 | 0.49 | 0.24 – 0.95 | **0.045** | 0.51 | 0.24 – 0.97 | 0.053 | 0.51 | 0.24 – 0.97 | 0.054 |
| Black or African American (not Hispanic or Latino) | 1.54 | 1.08 – 2.17 | **0.015** | 1.39 | 0.96 – 1.99 | 0.074 | 1.29 | 0.89 – 1.85 | 0.171 | 1.30 | 0.90 – 1.87 | 0.159 |
| Hispanic or Latino | 1.33 | 0.95 – 1.84 | 0.091 | 1.18 | 0.83 – 1.65 | 0.348 | 1.11 | 0.78 – 1.55 | 0.563 | 1.11 | 0.78 – 1.56 | 0.542 |
| *Household Income (Ref: < $50,000)* |  |  |  |  |  |  |  |  |  |  |  |  |
| $100,000+ | 1.42 | 1.07 – 1.89 | **0.015** | 1.63 | 1.20 – 2.21 | **0.002** | 1.42 | 1.04 – 1.94 | **0.028** | 1.28 | 0.74 – 2.17 | 0.360 |
| $50,000-$100,000 | 1.05 | 0.81 – 1.36 | 0.721 | 1.18 | 0.90 – 1.54 | 0.224 | 1.10 | 0.84 – 1.44 | 0.493 | 1.07 | 0.70 – 1.63 | 0.767 |
| *Region (Ref: South)* |  |  |  |  |  |  |  |  |  |  |  |  |
| Midwest | 0.93 | 0.70 – 1.23 | 0.608 | 1.02 | 0.76 – 1.36 | 0.901 | 1.06 | 0.79 – 1.42 | 0.702 | 1.06 | 0.79 – 1.42 | 0.695 |
| Northeast | 0.90 | 0.67 – 1.22 | 0.517 | 0.96 | 0.70 – 1.32 | 0.811 | 0.97 | 0.71 – 1.33 | 0.872 | 0.98 | 0.71 – 1.34 | 0.887 |
| West | 0.81 | 0.60 – 1.09 | 0.176 | 0.85 | 0.62 – 1.16 | 0.317 | 0.88 | 0.64 – 1.19 | 0.401 | 0.88 | 0.64 – 1.19 | 0.406 |
| *Community Type (Ref: Suburban)* |  |  |  |  |  |  |  |  |  |  |  |  |
| Rural | 1.43 | 1.07 – 1.90 | **0.016** | 1.50 | 1.11 – 2.03 | **0.008** | 1.59 | 1.17 – 2.16 | **0.003** | 1.59 | 1.17 – 2.16 | **0.003** |
| Urban | 2.27 | 1.78 – 2.89 | **<0.001** | 1.96 | 1.52 – 2.52 | **<0.001** | 1.92 | 1.49 – 2.48 | **<0.001** | 1.91 | 1.49 – 2.47 | **<0.001** |
| *Dog/Cat Ownership (Ref: Acquired Animals Before March 2020 But Not Since)* |  |  |  |  |  |  |  |  |  |  |  |  |
| Animals Acquired Before March 2020 and During Pandemic | 6.74 | 5.34 – 8.56 | **<0.001** | 5.08 | 3.98 – 6.52 | **<0.001** | 4.80 | 3.75 – 6.17 | **<0.001** | 4.79 | 3.75 – 6.16 | **<0.001** |
| Animals Acquired During Pandemic But Not Before | 3.03 | 1.75 – 5.05 | **<0.001** | 2.99 | 1.68 – 5.10 | **<0.001** | 3.02 | 1.70 – 5.17 | **<0.001** | 3.02 | 1.70 – 5.17 | **<0.001** |
| *Concerns (Ref: No Concern or Low Concern)* |  |  |  |  |  |  |  |  |  |  |  |  |
| Worried about Employment and Job Security |  |  |  | 1.38 | 1.02 – 1.86 | **0.038** | 1.34 | 0.99 – 1.81 | 0.060 | 1.34 | 0.99 – 1.81 | 0.059 |
| Worried May Not Be Able to Stay in Home |  |  |  | 1.91 | 1.43 – 2.55 | **<0.001** | 1.84 | 1.38 – 2.46 | **<0.001** | 1.83 | 1.37 – 2.45 | **<0.001** |
| Worried about Financial Security |  |  |  | 1.04 | 0.76 – 1.41 | 0.825 | 1.05 | 0.78 – 1.44 | 0.738 | 1.05 | 0.77 – 1.43 | 0.743 |
| Worried about Ability to Afford Veterinary Care |  |  |  | 1.75 | 1.28 – 2.41 | **0.001** | 1.79 | 1.30 – 2.46 | **<0.001** | 1.78 | 1.30 – 2.45 | **<0.001** |
| Worried Animal Will Have Behavior Problems |  |  |  | 1.38 | 1.02 – 1.87 | **0.036** | 1.29 | 0.95 – 1.75 | 0.099 | 1.29 | 0.95 – 1.75 | 0.102 |
| Worried Not As Much Time to Care for and Spend Time with Animal |  |  |  | 1.09 | 0.81 – 1.46 | 0.580 | 1.03 | 0.76 – 1.38 | 0.853 | 1.03 | 0.77 – 1.39 | 0.847 |
| Worried Animal Will Limit Travel Ability |  |  |  | 1.33 | 1.02 – 1.74 | **0.039** | 1.29 | 0.98 – 1.70 | 0.066 | 1.29 | 0.99 – 1.70 | 0.064 |
| *Working Status (Ref: Not Working from Home)* |  |  |  |  |  |  |  |  |  |  |  |  |
| Working from Home |  |  |  |  |  |  | 1.90 | 1.47 – 2.45 | **<0.001** | 1.80 | 1.21 – 2.67 | **0.004** |
| *Household Income x Working Status (Ref: Income < $50,000 and Not Working from Home)* |  |  |  |  |  |  |  |  |  |  |  |  |
| Income > $100,000 and Working from Home |  |  |  |  |  |  |  |  |  | 1.16 | 0.63 – 2.18 | 0.643 |
| Income $50,000-$1000,000 and Working from Home |  |  |  |  |  |  |  |  |  | 1.06 | 0.62 – 1.82 | 0.839 |
| Observations | 5531 | | | 5531 | | | 5531 | | | 5531 | | |
| -2LL | 2482.8 | | | 2286.8 | | | 2262.1 | | | 2261.9 | | |
| AIC | 2516.8 | | | 2334.8 | | | **2312.1** | | | 2315.9 | | |
| R^2^ Tjur | 0.226 | | | 0.290 | | | 0.300 | | | 0.300 | | |
