# Supplementary material for: Characterizing Pet Acquisition and Retention During the COVID-19 Pandemic
Source: Front Vet Sci. 2021 Nov 18;8:781403. doi: 10.3389/fvets.2021.781403 (PMC8637628; doi:10.3389/fvets.2021.781403)
Supplement: Supplementary file 3 [file Table_3.docx]

**[US OMNI]**

**[ASPCA-COVID PET ACQUISITIONS]**

**[ADAM LIFSON]**

**[GENPOP1]**

**[THIS STUDY NEEDS N=1,005 TO GVF1]**

**[REPEAT WAVE 869-FRIDAY WITH NO CHANGES]**

**[THIS IS DA APPROVED]**

GVF1. Which of these animals (dog, cat, or equine) did you own before the pandemic (before March 2020)?  Please select all that apply.

**[RANDOMIZE ANCHOR I DID NOT]**

Dog

Cat

Equine (Horse/donkey/mule)

I did not own a dog, cat, or equine before the pandemic **[VALIDATE]**

GVF2. Which of these animals (dog, cat, or equine) do you now own (as of May 2021)?  Please select all that apply.

**[RANDOMIZE ANCHOR I DO NOT]**

Dog

Cat

Equine (Horse/donkey/mule)

I do not own a dog, cat, or equine **[VALIDATE]**

GVF3. Have you rehomed (or given up) a pet since March 2020? Please select all that apply.

**[RANDOMIZE ANCHOR NO AND NOT SURE]**

Yes, I rehomed a dog.

Yes, I rehomed a cat.

Yes, I rehomed a horse/donkey/mule.

No **[VALIDATE]**

Not sure **[VALIDATE]**

GVF4. Are you considering rehoming (or giving up) an animal in the near future (next 3 months)? Please select all that apply.

**[RANDOMIZE ANCHOR NO AND NOT SURE]**

Yes, I am considering rehoming a dog.

Yes, I am considering rehoming a cat.

Yes, I am considering rehoming a horse/donkey/mule.

No **[VALIDATE]**

Not sure **[VALIDATE]**

GVF5. Have you acquired a dog, cat, or horse, or fostered a pet since the start of the pandemic (between March 2020 and May 2021)? Please select all that apply.

My household got a dog

My household got a cat

My household got a horse/donkey/mule

I fostered a dog, cat, or equine sometime between March 2020 to May 2021

I have neither acquired a pet nor fostered during that time. **[VALIDATE]**

**IF “code 1-3” TO GVF1 OR “code 1-3” TO GVF2 OR “code 1-3” TO GVF5, CONTINUE; ELSE SKIP TO NEXT SECTION**

**[ASK GVF6-GVF7 IF CODE 1 (dog) AT GVF5]**

GVF6. From where did you get your dog during the pandemic? (if you got more than one animal, please answer for the animal whose name starts with the letter that comes first in the alphabet)

**[SINGLE SELECT]**

from a breeder (I saw in person where the dog was raised)

from a breeder (I met the breeder somewhere or had the dog transported to me)

from a pet store (PURCHASED)

from a shelter or rescue organization

from an individual, friend, family member or neighbor (PURCHASED)

from an individual, friend, family member or neighbor (FOR FREE)

Other – please specify **[INSERT TEXT BOX]**

GVF7. Is the dog you acquired during the pandemic still with you?

**[SINGLE SELECT]**

Yes

No, they are with a friend, family, or neighbor

No, they are at a shelter or rescue

No, I sold my animal

No, my animal died

No, my animal got lost

Other- please specify **[INSERT TEXT BOX]**

**[ASK GVF8-GVF9 IF CODE 2 (cat) AT GVF5]**

GVF8. From where did you get your cat during the pandemic? (if you got more than one cat, please answer for the animal whose name starts with the letter that comes first in the alphabet)

**[SINGLE SELECT]**

from a breeder (I saw in person where the cat was raised)

from a breeder (I met the breeder somewhere or had the cat transported to me)

from a pet store (PURCHASED)

from a shelter or rescue organization

from an individual, friend, family member or neighbor (PURCHASED)

from an individual, friend, family member or neighbor (FOR FREE)

Other – please specify **[INSERT TEXT BOX]**

GVF9. Is the cat you acquired during COVID still with you?

**[SINGLE SELECT]**

Yes

No, they are with a friend, family, or neighbor

No, they are at a shelter or rescue

No, I sold my cat

No, my cat died

No, my cat got lost

Other- please specify **[INSERT TEXT BOX]**

**[ASK GVF10-GVF11 IF CODE 3 (EQUINE) AT GVF5]**

GVF10. From where did you get your equine during the pandemic? (if you got more than one equine, please answer for the animal whose name starts with the letter that comes first in the alphabet)

**[SINGLE SELECT]**

from a breeder (I went to the facility)

from a breeder (I did not go to the facility)

from a shelter or rescue

from an auction

from a kill pen

from a broker

from a trainer

from an individual, friend, family member or neighbor (PURCHASED)

from an individual, friend, family member or neighbor (FOR FREE)

Other – please specify **[INSERT TEXT BOX]**

GVF11. Is the equine you acquired during COVID still with you?

**[SINGLE SELECT]**

Yes

No, they are with a friend, family, or neighbor

No, they are at a shelter or rescue

No, I sold my horse/donkey/mule

No, my horse/donkey/mule died

Other- please specify **[INSERT TEXT BOX]**

**[ASK GVF12 IF CODES 1-3 AT GVF5]**

GVF12. Were you already planning on acquiring a new animal during this timeframe (March 2020 through May 2021)?

Yes – before the pandemic began, I was already planning on acquiring a new animal

No – I decided to get a new animal after the pandemic had begun

**[ASK ALL]**

GVF13. Please select the following answer choice that best describes your current work status.

**[SINGLE SELECT]**

I am currently employed and working fully remotely, temporarily

I am currently employed and working fully remotely, permanently

I am currently employed and working partly remotely partly away from home

I am currently employed and working fully away from home

I am retired

I don’t need to work

I am currently unemployed

other

**[ASK ALL]**

GVF14. As COVID restrictions are lifted, how concerned are you about the following statements (rate your concern on a scale of 1-5; 5 being extremely concerned and 1 being not at all concerned).

**[GRID ACROSS: PROGRESSIVE]**

5- Extremely concerned

4

3

2

1- Not at all concerned

**[GRID DOWN: RANDOMIZE]**

I won't have as much time to care for and/or spend with my animal.

My animal will have behavior problems as a result of a change in schedule.

I’d like to travel more and I feel limited by my animal.

I’m worried about being able to afford veterinary care for my animal.

I’m worried about my employment and job security.

I’m worried about my financial security.

I’m worried that I may not be able to stay in my home.
